# Comparison of early and late postoperative outcomes between chordal reconstruction and quadrangular resection in patients with posterior mitral valve prolapse: a single-center retrospective study

**DOI:** 10.1186/s12872-022-03010-z

**Published:** 2022-12-17

**Authors:** Xiangwei Song, Qingsong Wu, Guican Zhang, Xiaofu Dai, Feng Lin, Liangwan Chen, Qimin Wang

**Affiliations:** 1 Department of Cardiovascular Surgery, Union Hospital, Fujian Medical University, Fuzhou, 350001 Fujian People’s Republic of China; 2 Fujian Medical University, Fuzhou, 350001 Fujian People’s Republic of China; 3 Key Laboratory of Cardio-Thoracic Surgery, Fujian Medical University, Fujian Province University, Fuzhou, 350001 Fujian People’s Republic of China

**Keywords:** Posterior mitral valve prolapse, Chordal reconstruction, Quadrangular resection, Postoperative hemolysis, Recurrent mitral regurgitation, Left ventricular end-diastolic diameter

## Abstract

**Background:**

To compare the early and late postoperative outcomes of chordal reconstruction (CR) and quadrangular resection (QR) in patients with posterior mitral valve prolapse (PMPL).

**Methods:**

Between January 2008 and December 2018, 305 patients with PMPL who underwent mitral valve plasty (MVP) were included in this retrospective analysis. The CR and QR procedures were performed in 169 patients (CR group) and 136 patients (QR group), respectively. Early and late postoperative outcomes were compared between the groups.

**Results:**

Follow-up was complete in 96.4% (294/305) of patients, with a mean follow-up of 81.2 ± 30.4 months. No 30-day mortality was observed in any of the patients. The success rate of the mitral valve repair was similar in both groups (99.4% vs. 98.5%, *P* = 0.850). The incidence of early postoperative hemolysis was lower in the CR group than in the QR group (0.00% vs. 3.0%, *P* = 0.024). Postoperative left ventricular end-diastolic diameter (LVEDD) decreased more significantly in the CR group than in the QR group at 3 months (8.15 [1.30,12.65] vs. 3.25 [− 0.05, 8.75] mm, *P* < 0.001). During follow-up, the overall survival rates were 95.1% and 94.6% in the CR and QR groups, respectively. The incidence of reoperation for moderate or severe mitral regurgitation (MR) was similar in both groups (4.3% vs.5.4%, *P* = 0.653), but the time interval between the initial operation and reoperation was shorter in the QR group than in the CR group (84.3 ± 36.1 vs. 120.9 ± 27.6 months, *P* = 0.026). The LVEDD enlargement was more significant in the QR group than in the CR group (4.5 [3.6, 4.5] vs. 2.4 [1.3, 2.8] mm, *P* < 0.001).

**Conclusion:**

CR and QR are effective techniques for patients with PMPL. Both techniques resulted in a low incidence of recurrent MR. However, CR can reduce early postoperative hemolysis and LVEDD more significantly. During the long-term follow-up, reoperations due to recurrent MR were performed at a longer interval after the initial operation. LVEDD expansion was better avoided in the CR group.

## Background

Mitral valve plasty (MVP) is the gold standard treatment for mitral valve prolapse owing to its ability to produce good long-term results [1, 6] MVP preserves the mitral leaflet and subvalvular structures to better preserve or restore normal leaflet movement and increase the mitral valve leaf surface of the coaptation 7. In addition, it maintains the connection between the papillary muscle of the chordae tendineae and the left ventricle in combination with a ring annuloplasty to stabilize the original mitral valve ring 4. Thus, the MVP is important in preventing further left ventricular dilation and maintaining postoperative left ventricular systolic function 8. For more than half a century, MVP has been developed using many different surgical techniques [9, 16] in the pastdecade, the most common techniques used in our hospital for the treatment of posterior mitral valve prolapse (PMPL) were chordal reconstruction (CR) and quadrangular resection (QR) procedures. However, systematic evaluation of the early and late postoperative outcomes of the two procedures is lacking.

Therefore, we aimed to retrospectively investigate the early and late postoperative outcomes of CR and QR in patients with PMPL.

## Methods

### Study population

This single-center study evaluated 305 Chinese patients who presented to our hospital between January 2008 and December 2018 and were eligible for inclusion in this retrospective study. We included patients who: (i) were diagnosed with nonrheumatic isolated PMPL confirmed by echocardiography or transesophageal echocardiography (TEE) and (ii) had only undergone surgery with CR or QR. We excluded patients if they (i) had a history of surgery for mitral valve disease, (ii) were diagnosed with mitral valve prolapse (MVL) due to ischemic cardiomyopathy or endocarditis, (iii) were diagnosed with mitral stenosis and congenital mitral valve disease, and (iv) had anterior or bileaflet prolapse.

This retrospective study was approved by the ethics committee of Union Hospital of Fujian Medical University and conformed to the Declaration of Helsinki. The requirement for informed consent was waived owing to the retrospective nature of the study.

### Clinical data

The data collected included baseline characteristics, preoperative conditions, operative details, postoperative outcomes, and follow-up data. Baseline characteristics included sex, age, body mass index, heart function classification (NYHA), acute left heart failure, hydrothorax, coronary heart disease, hypertension, diabetes mellitus, atrial fibrillation, chronic obstructive pulmonary disease, left ventricular ejection fraction (LVEF), left atrial diameter, left ventricular end-diastolic/systolic diameter (LVEDD/LVESD), and mitral regurgitation (MR) grade. Operative details included operative approach, operative time, cardiopulmonary bypass (CPB) time, aorta-clamping time, and MVP failure. Postoperative outcomes included 30-day mortality and major adverse outcomes, including hemolysis, acute renal failure, gastrointestinal hemorrhage, multiple organ dysfunction, mechanical ventilation, intensive care in the ICU, and hospitalization time. Follow-up data included follow-up time, death, LVEF, left atrial diameter, LVED/LVESD, acute heart failure, chronic heart failure, new-onset atrial fibrillation, recurrent MR (+++/++++), reoperation for recurrent MR, and the interval between the initial operation and reoperation. Postoperative hemolysis was defined by progressive anemia and dark urine after MVP in a patient with no history of hemolysis before MVP, and the observation of residual mitral regurgitation byechocardiography [17].

### Operative technique

Intraoperatively, all patients were under general anesthesia, and TEE was routinely performed. TEE confirmed the location and extent of MPL. Thoracoscopic or conventional median sternal incision or partial incision of the lower sternum was used to access the pericardial cavity. After heparinization with 3 mg/kg heparin sodium, CPB was established when the activated clotting time of whole blood was > 480 s. When the nasopharyngeal temperature dropped to 30–32℃, the aorta was cross-clamped, and myocardial protective cardioplegia fluid was injected through the ascending aortic root perfusion tube. Carbon dioxide was used to flood the surgical field. The left atrium was accessed via the atrial septum or atrioventricular sulcus, and the mitral valve was exposed, with a detailed examination of the mitral valve, subvalvular structure, and papillary muscles. Valve competency was tested using a bulb syringe, and the location and extent of MPL were determined. CR or QR procedure was then performed.

The CR procedure was performed using artificial chordae tendineae Gore-tex sutures (W. L. Gore & Associates, Inc, Elkton, Maryland) sewn through the posterolateral papillary muscle 16. The artificial chordae were brought up and sewn through the prolapsed posterior mitral valve a few millimeters from the edge of the valve. Multiple artificial chordae were used when necessary. Valve competency was tested by filling the left ventricle with a saline solution using a bulb syringe to determine the length of the chordae. With the length of the chordae adjusted, a titanium clip was placed on the chordae to avoid sliding the knot while tying the chordae. The valve competency was again tested with a bulb syringe. If the result of the valve competency was satisfactory, the mitral valvuloplasty ring (BalMedic, Beijing Bairen Medical Technology Co., Ltd. Beijing), Edwards (Lifesciences Corp., Irvine, CA, USA), St Jude (St. Jude Medical, St. Paul, Minnesota, USA), or Medtronic (Medtronic Inc. Minneapolis, Minnesota, USA) was selected according to the size of the measured annulus and was sutured to the annulus.

The QR procedure adopted the techniques described in Carpentier’s report after careful examination of the diseased valve 4. Valve repair proceeded with QR of the prolapsed portion of the posterior leaflet with the corresponding annular plication, followed by suture reapproximation of the free leaflet edges. The valve competency was tested, and the mitral valvuloplasty ring was sutured to the annulus. The surgical incision was closed, the cross-clamped ascending aorta was opened, and the heart resumed beating. Valve competency was reassessed by TEE after CPB was stopped. If the results were satisfactory, the operation was completed. If the valve competency was unsatisfactory, or the mitral valve still had moderate or more regurgitation, then re-repair surgery was conducted.

### Follow-up

After discharge, patients were followed-up in the outpatient clinic, follow-up room, through telephone, or WeChat, and warfarin was administered orally every day for 3 months. Before discharge and 3 months after surgery, patients underwent transthoracic echocardiography, which was then performed annually. The patients were followed-up until December 2021.

### Statistical analysis

Data are presented as numbers or percentages for categorical variables, whereas median and interquartile range or mean ± standard deviation were used for continuous variables according to whether they followed a Gaussian distribution. T-test or Mann–Whitney U test for continuous variables and χ2 test for categorical variables. The survival curve was determined using the Kaplan–Meier curve and log-rank test to assess group differences. The analysis was performed using SPSS version 19.0 (IBM Corp., Armonk, NY, USA). The threshold for significance was set at *P* < 0.05.

## Results

In total, 305 patients underwent CR (CR group, n = 169) or QR (QR group, n = 136) procedures in this study. There were no statistical differences in the baseline characteristics and preoperative data, as shown in Table 1.

**Table 1:** Preoperative data of the two patient groups

Valuables	Group CR (n = 169)	Group QR (n = 136)	*P* value
Age (years)	54.0 (45.0, 61.0)	56.0 (47.0, 62.0)	0.235
Male gender (%)	110 (65.1)	92 (67.7)	0.639
Body mass index (kg/m^2^)	21.3 (19.5, 23.9)	21.9 (20.0, 24.7)	0.087
Heart function classification (NYHA)			
I (%)	5 (3.0)	4(2.9)	0.740
II (%)	35(20.7)	27 (19.9)	0.853
III (%)	113 (66.9)	94 (69.1)	0.675
IV (%)	16 (9.5)	11 (8.1)	0.673
Acute left heart failure (%)	5 (3.0)	7 (5.2)	0.328
Hydrothorax (%)	2 (1.2)	4 (2.9)	0.494
Coronary heart disease (%)	10 (5.9)	7 (5.2)	0.771
Hypertension (%)	29 (17.2)	33 (24.3)	0.125
Diabetes mellitus (%)	14 (8.3)	17 (12.5)	0.226
Atrial fibrillation (%)	4 (2.37)	4 (2.9)	0.961
Left atrial diameter (mm)	42.8 (36.8, 48.8)	43.2 (37.1, 49.2)	0.901
LVEDD (mm)	54.8 (51.4, 60.1)	54.1 (50.6, 58.5)	0.331
LVESD (mm)	33.8 (31.0, 35.8)	33.0 (30.3, 35.5)	0.541
MR grade			
+++ (%)	22 (13.0)	15 (11.0)	0.597
++++ (%)	147 (87.0)	121 (89.0)	0.597
LVEF (%)	69.1 (64.4, 73.2)	69.1 (64.9, 72.5)	0.720

Numbers or percentages for categorical variables, median and interquartile range or mean ± standard deviations for continuous variables. T test or MannWhitney U test for continuous variables and χ2 test for categorical variables

### Operative data

The operative details of the two groups are summarized in Table 2. There was no statistical difference in the operative details between the two groups. Mitral valvuloplasty rings were used in all patients who underwent MVP. There was no significant difference between the two groups with different manufacturers, shapes, and sizes of mitral valvuloplasty rings. The success rate of mitral valve repair was similar in both groups (99.4% vs. 98.5%, *P* = 0.850).

**Table 2:** Surgical data of the two patient groups

Valuables	Group CR (n = 169)	Group QR (n = 136)	*P* value
Operative approach			
Thoracoscopic (%)	12 (7.1)	10 (7.4)	0.933
Median sternal incision (%)	120 (71.0)	95 (69.9)	0.826
Partial sternal incision (%)	37 (21.9)	31 (22.8)	0.851
Operative time (min)	180.0 (165.0, 195.0)	180.0 (166.5, 190.0)	0.786
CPB time (min)	81.0 (70.0, 94.0)	84.0 (76.0, 90.0)	0.753
Aorta clamped time (min)	30.0 (26.0, 36.0)	30.0 (26.0, 33.0)	0.143
Mitral valvuloplasty ring			
BalMedic (%)	115 (68.0)	83 (61.0)	0.202
St.Jude (%)	29 (17.2)	33 (24.3)	0.125
Edwards	17 (10.2)	13 (9.6)	0.884
Medtronic (%)	8 (4.7)	7 (5.2)	0.868
MVP failed (%)	1 (0.6)	2 (1.5)	0.850
MVP success rate (%)	168 (99.4)	134 (98.5)	0.850
Artificial chordae number	1.47 ± 0.52	N/A	N/A

Numbers or percentages for categorical variables, median and interquartile range or mean ± standard deviations for continuous variables. T test or MannWhitney U test for continuous variables and χ2 test for categorical variables

### Early mortality and morbidity

Postoperative hemolysis occurred in four patients in the QR group but not in the CR group (3.0% vs. 0.00%, *P* = 0.024). Two patients showed remission of hemolysis after conservative treatment, and two underwent reoperation to replace the mitral valve. Postoperative LVEDD decreased more significantly in the CR group than in the QR group at 3 months (8.2 [1.3,12.7] vs. 3.3 [− 0.1, 8.8] mm, *P* < 0.001). All the patients recovered and were discharged without any complications requiring treatment. The postoperative complications and morbidity data are summarized in Table 3.

**Table 3:** Postoperative early outcomes data of the two patient groups

Valuables	Group CR (n = 168)	Group QR (n = 134)	*P* value
Early death (%)	0 (0.0)	0 (0.0)	N/A
Hemolysis (%)	0 (0.0)	4 (3.0)	0.024
Acute renal failure (%)	1 (0.6)	2 (1.5)	0.844
Left atrial diameter (mm)	39.6 (33.6, 44.8)	40.0 (34.4, 43.8)	0.996
LVEF (%)	69.1 (65.9, 73.0)	69.1 (65.3, 71.3)	0.319
LVEDD decreased (mm)	8.2 (1.3,12.7)	3.3 (− 0.1, 8.8)^a^	< 0.001
LVESD decreased (mm)	2.50 (1.1, 3.1)	1.35 (− 1.4, 4.8)^a^	0.089
Gastrointestinal hemorrhage (%)	2 (1.2)	1 (0.8)	0.844
Multiple organ dysfunction (%)	1 (0.6)	1 (0.8)	0.580
Mechanical ventilation (h)	5.0 (4.0, 7.0)	5.0 (4.0, 8.0)	0.515
Intensive care in ICU (h)	17.0 (15.0, 19.0)	17.0(16.0, 20.0)	0.325
Hospitalization time (d)	9.0 (8.0, 12.0)	10.0 (8.0, 13.0)	0.258

Numbers or percentages for categorical variables, median and interquartile range or mean ± standard deviations for continuous variables. T test or MannWhitney U test for continuous variables and χ2 test for categorical variables

^a^Negative numbers means decrease

### Follow-up and late outcomes

**Fig. 1:**
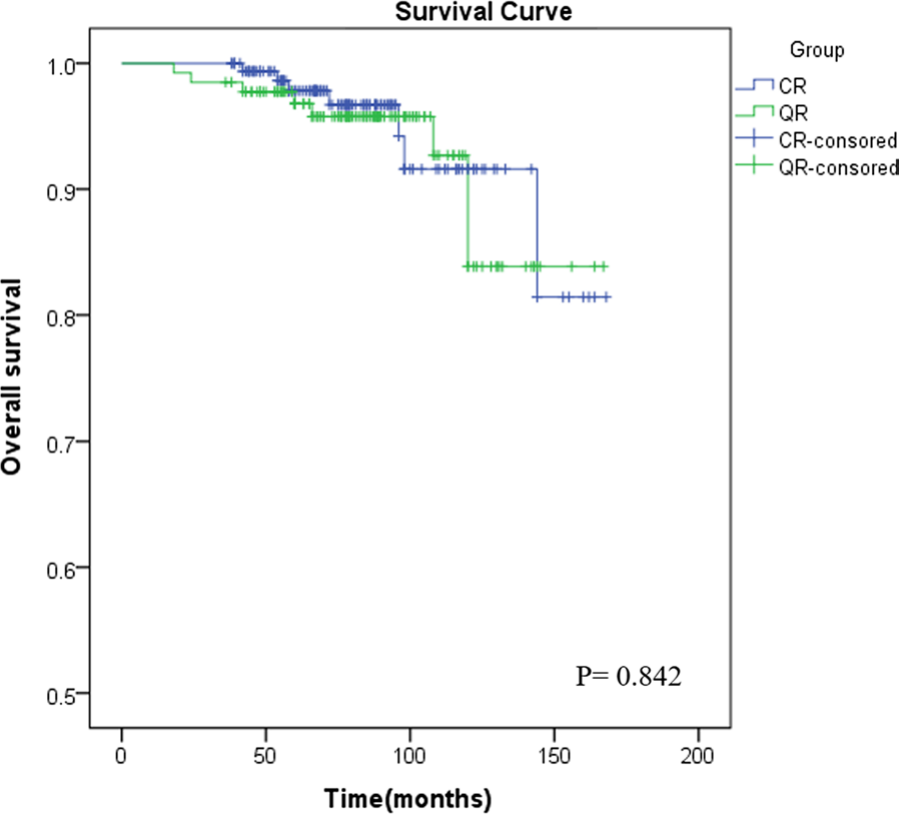
Survival curve

Follow-up was complete in 96.4% (294/305) of the patients (eight patients were lost to follow-up, and three were converted to intermediate mitral valve replacement) with a mean follow-up of 78.7 ± 30.3 and 84.7 ± 30.4 months, respectively. The overall survival rates of the CR and QR groups were 95.1% and 94.6%, respectively. Survival curves are shown in Fig. 1. The incidence of reoperation for moderate or severe recurrent MR was similar in both groups (4.3% vs. 5.4%, *P* = 0.653). However, the time interval between the initial operation and reoperation was significantly shorter in the QR group than in the CR group (84.3 ± 36.1 vs. 120.9 ± 27.6 months, *P* = 0.026). LVEDD enlargement was more significant in the QR group than in the CR group (4.5 [3.6, 4.5] mm vs. 2.4 [1.3, 2.8] mm, *P* < 0.001). There was no significant difference in new-onset atrial fibrillation between the two groups (7.4% vs. 10.9%, *P* = 0.298). In addition, the two groups had no significant difference in the incidence of acute heart failure (1.2% vs. 1.6%, *P* = 0.787) or chronic heart failure (2.5% vs. 4.7%, *P* = 0.483) (Tables 4 and 5).

**Table 4:** Follow-up data of the two patient groups

Valuables	Group CR (n = 163)	Group QR (n = 129)	*P* value
Follow-up time (months)	78.7 ± 30.3	84.7 ± 30.4	0.092
Follow-up death (%)	8 (4.9)	7 (5.4)	0.842
Overall survival rates (%)	155 (95.1)	122 (94.6)	0.842
LVEF (%)	67.6 (64.4,71.2)	66.9 (63.9, 70.3)	0.308
LVEDD dilated (mm)	2.4 (1.3, 2.8)	4.5 (3.6, 4.5)	< 0.001
LVESD dilated (mm)	2.0 (1.3, 2.5)	2.3 (1.2, 2.4)	0.383
Acute heart failure (%)	2 (1.2)	2 (1.6)	0.787
Chronic heart failure (%)	4 (2.5)	6 (4.7)	0.483
New-onset atrial fibrillation (%)	12 (7.4)	14 (10.9)	0.298
recurrent MR (+++/++++) (%)	7 (4.3)	8 (6.2)	0.463
reoperation for recurrent MR (%)	7 (4.3)	7 (5.4)	0.653

Numbers or percentages for categorical variables, median and interquartile range or mean ± standard deviations for continuous variables. T test or MannWhitney U test for continuous variables and χ2 test for categorical variables

**Table 5:** Interval between the initial operation and reoperation time of the two patient groups for recurrent MR

Valuables	Group CR (n = 7)	Group QR (n = 7)	*P* value
interval between the initial operation and reoperation (months)	120.9 ± 27.6	84.3 ± 36.1	0.026

Mean ± standard deviations and T test for continuous variables

## Discussion

Currently, MVP is the first choice of treatment for severe mitral insufficiency caused by PMPL. CR and QR procedures have been proven to have good short- and long-term outcomes [18, 19].

Postoperative hemolysis is a serious complication of MVP and is dangerous and even life-threatening. It usually occurs during the early stages of MVP [20, 21]. In this study, we found that in the early stages, the incidence of postoperative hemolysis was significantly higher in the QR group than in the CR group. An important finding of our study was that the median interval between MVP and hemolysis was very short. Therefore, it is reasonable to assume that hemolysis after MVP is surgery related. We found that all patients with postoperative hemolysis had a small eccentric regurgitant blood flow with high shear stress. We analyzed intraoperative TEE to identify patients at risk of such a complication; however, we encountered difficulties in conducting further analysis and research owing to the small number of positive cases.

According to our analysis, the cause of postoperative hemolysis is related to the MVP technology. In the QR group, the cutting and sewing technique, with suture between the two cutting margins after QR of the mitral leaf tissue was required. When the suture was not tight or the suture tension was too high, the suture margins tore and created small gaps, resulting in small eccentric regurgitant blood flow with high shear stress, thereby leading to hemolysis [22, 23]. Alternatively, there may be small protrusions in the suture margin when the posterior leaf is resected and resutured, which may not fit the anterior valve well when the valve is closed, or there may be small folds after the anastomosis. This, again, could result in a small eccentric regurgitant blood flow with high shear stress, leading to hemolysis. However, in the CR group, the procedure did not destroy the original structure of the leaflets, and it was difficult to produce small gaps and eccentric regurgitant blood flow. The fractured original chordae tendineae were replanted and restored to their original physiological state, and the anterior and posterior leaflets were completely combined. Therefore, hemolysis can be avoided to an extent, and when small regurgitation of blood flow appears, they also present with central regurgitation. Therefore, more attention should be paid to the direction and source of regurgitation for some small regurgitation blood flow in the TEE examination before the end of surgery.

Left ventricular enlargement is a common cause of MR, and its pathogenesis is complex. Mitral valve regurgitation increases the volume load of the left ventricle, leading to left ventricular remodeling and resulting in downward and apex displacement of the papillary muscle. Increased mitral valve traction can lead to increased left ventricular papillary muscle tone, lengthening, or even rupture of chordae tendineae, causing acute or chronic heart failure. In addition, myocardial contractility is weakened, and left ventricular dilation and mitral valve annular dilation are observed. This eventually leads to incomplete valve closure and MR [24, 26]. In this study, we found that the left ventricular diameter decreased to varying degrees in all patients after MVP, and the reduction was greater in the CR group. During the CR procedure, artificial chordae tendineae were used to retract the prolapsed leaflets and fixed on the original left ventricular papillary muscles, which maintained the original left ventricular function to the maximum extent, preserved the subvalvular structure, and maintained the continuity of the mitral valve device. This played an important role in maintaining the left ventricular function 27. In addition, the artificial chordae tendineae has a traction effect on the papillary muscles; therefore, it has a certain effect on preventing left ventricular dilatation 28. Even when recurrent MR occurs at later follow-up, the rate of left ventricular remodeling is limited, preventing left ventricular enlargement within a short period.

The key to the CR procedure is determining the length of the artificial chordae tendineae. Chordae tendineae with lengths that are too long or too short will cause incomplete valve closure and MR [29, 30]. Our experience was based on the lengths of the chordae tendineae and the anterior and posterior mitral leaflet closure lines. If necessary, methylene blue was used to identify the height and surface of the anterior and posterior mitral leaflets. After adjusting the length of the artificial chordae tendineae, they were temporarily fixed with titanium clips. A water injection test was performed to observe the closure of the mitral leaflets.

The QR procedure can also reduce MR and left ventricular afterload, such that the left ventricle diameter can be reduced, which will reduce the area of the posterior mitral leaflet tissue, resulting in the reduction of the height and area of anterior and posterior mitral leaflets. Our experience in processing was carefully comparing and measuring the leaflet with an appropriate excision area. The width of the posterior mitral leaflet is generally 10–20 mm. To ensure good valve competency, the heights of the anterior and posterior mitral leaflets should be kept at > 12 mm. Finally, both methods use a mitral valvuloplasty ring to restore the normal size and shape of the annulus, prevent further expansion of the mitral annulus, increase the junction surface of the anterior and posterior mitral leaflets, and increase the durability of the mitral valve [31]. However, in some patients with chronic left ventricular enlargement, the myocardium of the left ventricle undergoes remodeling, leading to failure of the left ventricle to recover its original physiological state. Another problem is that there may be too much resection area in the QR procedure, which may lead to anterior movement of the valvular anastomosis line and systolic anterior motion, resulting in left ventricular outflow tract obstruction [32]. Although this phenomenon was not observed in this study, its occurrence must always be carefully avoided.

Based on our previous analysis, QR techniques reduced the junction surface of the anterior and posterior mitral leaflets; therefore, they were also tested for valve durability, which was also confirmed in our study. Although the incidence of reoperation for recurrent MR was similar between the two groups, the interval between the first operation and reoperation was shorter in the QR group than in the CR group. For MR, the surface of the anterior and posterior mitral leaflets junction is the key factor, and recurrent MR after MVP is mostly caused by the lack of the anterior and posterior mitral leaflets junction. In patients with acute chordae tendineae rupture, the QR method may result in insufficient posterior mitral leaflet area, poor mitral anterior and posterior mitral leaflets fit, or very high tension of the suture line at the edge of the posterior mitral valve. However, artificial chordae tendineae can avoid this defect [33]. The QR technique provided good height and area of the anterior and posterior mitral leaflets junction, and the surgical effect was satisfactory. In the long-term follow-up, the left ventricular diameter expanded again, causing a decline in height and area of the anterior and posterior mitral leaflets junction, resulting in a gradual increase in recurrent MR, which in turn affected the further expansion of the left ventricle diameter. This was also confirmed by the results of LVEDD in both groups during the long-term follow-up in our study.

### Limitations

This was a single-center, retrospective study with a small number of patients. Therefore, a multicenter study with larger sample size is needed. Only patients with posterior mitral valve prolapse were included in this study. However, many patients with anterior mitral valve prolapse or bilateral mitral valve prolapse are also involved in practical applications; hence, further controlled studies are required. Simultaneously, our hospital uses not only CR and QR procedures but also other techniques such as edge-to-edge, chordae tendineae transfer, and valve leaflet folding. However, these procedures were not included in this study because of the small number of cases, which may have biased the results.

## Conclusion

The early and long-term outcomes of the CR and QR techniques for the treatment of PMPL were satisfactory and proved reliable, reproducible, and durable. However, in our retrospective controlled study, the CR procedure reduced LVEDD more significantly because it reduced early postoperative hemolysis. In the long-term follow-up, the CR procedure was more recognized because of the longer interval between the initial operation and reoperation and better avoidance of LVEDD dilation.

## Data Availability

The datasets used and/or analysed during the current study are available from the corresponding author on reasonable request.

## Abbreviations

CR: Chordal reconstruction
QR: Quadrangular resection
PMPL: Posterior mitral valve prolapse
MR: Mitral regurgitation
MVP: Mitral valve plasty
MPL: Mitral valve prolapse
TEE: Transesophageal echocardiography
LVEF: Left ventricular ejection fraction
LVEDD/LVESD: Left ventricular end-diastolic/systolic diameter
CPB: Cardiopulmonary bypass
